# Indoles induce metamorphosis in a broad diversity of jellyfish, but not in a crown jelly (Coronatae)

**DOI:** 10.1371/journal.pone.0188601

**Published:** 2017-12-27

**Authors:** Rebecca R. Helm, Casey W. Dunn

**Affiliations:** 1 Brown University, Providence, RI, United States of America; 2 Woods Hole Oceanographic Institution, Woods Hole, MA, United States of America; Tierarztliche Hochschule Hannover, GERMANY

## Abstract

Many animals go through one or more metamorphoses during their lives, however, the molecular underpinnings of metamorphosis across diverse species are not well understood. Medusozoa (Cnidaria) is a clade of animals with complex life cycles, these life cycles can include a polyp stage that metamorphoses into a medusa (jellyfish). Medusae are produced through a variety of different developmental mechanisms—in some species polyps bud medusae (Hydrozoa), in others medusae are formed through polyp fission (Scyphozoa), while in others medusae are formed through direct transformation of the polyp (Cubozoa). To better understand the molecular mechanisms that may coordinate these different forms of metamorphosis, we tested two compounds first identified to induce metamorphosis in the moon jellyfish *Aurelia aurita* (indomethacin and 5-methoxy-2-methylindole) on a broad diversity of medusozoan polyps. We discovered that indole-containing compounds trigger metamorphosis across a broad diversity of species. All tested discomedusan polyps metamorphosed in the presence of both compounds, including species representatives of several major lineages within the clade (Pelagiidae, Cyaneidae, both clades of Rhizostomeae). In a cubozoan, low levels of 5-methoxy-2-methylindole reliably induced complete and healthy metamorphosis. In contrast, neither compound induced medusa metamorphosis in a coronate scyphozoan, or medusa production in either hydrozoan tested. Our results support the hypothesis that metamorphosis is mediated by a conserved induction pathway within discomedusan scyphozoans, and possibly cubozoans. However, failure of these compounds to induce metamorphosis in a coronate suggests this induction mechanism may have been lost in this clade, or is convergent between Scyphozoa and Cubozoa.

## Introduction

Many animals from diverse groups go through one or more metamorphoses during their lives, drastically changing their physiology, morphology, ecology, and behavior [1,2]. This process of metamorphosis may be strikingly distinct between species, even when adult forms are similar. For example, the canonical life cycle in Medusozoa includes three life cycle stages: a swimming non-feeding planula larva, a long-lived benthic polyp, and a free-swimming medusa. Polyps are usually small and attached to a substrate, multiply asexually, and can live for years. When environmental conditions are conducive, polyps give rise to medusae. However, the method of medusae production is incredibly diverse.

In the Hydrozoa, medusae are produced through budding, where a small sphere of tissue grows from polyp tissue. Early buds are composed of an inner cellular mass, and an external cell layer. These different cell layers give rise to different components of the medusae (reviewed in [3] and [4]). In the Scyphozoa, the metamorphosis from polyp to medusa is termed strobilation. During strobilation, one or many constrictions form perpendicular to the polyp oral-aboral axis, and each ring of tissue develops into a juvenile medusa, termed an ephyra [5]. Strobilation is often triggered by an environmental cue, such as changes in temperature or salinity [6], or lunar cycles [7]. In contrast to both hydrozoans and scyphozoans, members of Cubozoa undergo complete or near-complete metamorphosis, where each polyp directly and fully transforms into a medusa, leaving little or no vestigial tissue behind [8–12]. The rare Staurozoa medusae metamorphose from the apical end of the polyp, and stay attached to the benthos [13]. This diversity in medusa formation is an important taxonomic character. For example, cubozoans were originally included in the clade Scyphozoa [14], but they were moved to their own group in 1973 when Werner discovered their unique form of metamorphosis [9].

Although the processes of medusa production are developmentally distinct between major clades, we know very little about the molecular mechanisms that initiate medusa formation within these groups. Recently, Kraus et al. [15] found that both a hydrozoan and scyphozoan show similar patterns of spatial gene expression for select homologues during medusa formation. It is possible that morphological differences in medusa production mask similarities at the cellular level. Several recent studies have found that both a subset of scyphozoans, and several cubozoans, metamorphosed in the presence of indoles [16–20]. However, these results have yet to be placed in an explicitly phylogenetic context, and indoles have yet to be tested on members of a key clade of Scyphozoa—the Coronatae. This makes evolutionary interpretation difficult.

To determine if indoles are effective at triggering metamorphosis in a species- or clade-specific manner, we tested two indole-containing compounds (indomethacin and 5-methoxy-2-methylindole) on a broad diversity of medusozoans. We discovered that indole-containing compounds trigger metamorphosis in all discomedusan scyphozoans tested, as well as one cubozoan species, but did not induce metamorphosis in the coronate polyps, or in two tested hydrozoans. We discuss the evolutionary implications for this pattern in relation to the evolution and development of medusa metamorphosis.

## Materials and methods

### Species selection, animal acquisition and care

We acquired a broad diversity of scyphozoan polyps to determine the phylogenetic breadth of indole efficacy within this group. Within Scyphozoa there are two major clades, Discomedusae and Coronatae. We sampled species of Discomedusae from multiple major lineages, including: Pelagiidae, Cyaneidae, Ulmaridae, Cepheidae, Cassiopeidae, and Mastigiidae. Discomedusan polyps are cultured in many public aquaria, and are readily available through marine labs. Coronate polyps, however, are not widely cultured, and are difficult to find in the wild. For this reason, we were only able to run trials on one species of Coronate polyp, *Linuche* sp. These same difficulties apply to cubozoans, which account for our small sample size for this clade. Within Hydrozoa, there are two major lineages, Hydroidolina and Trachylina [21,22]. We tested indoles on representatives from each of these clades.

Polyp cultures were obtained from multiple sources. Chris Doller and Steve Spina of the New England Aquarium provided cultures of *Chrysaora quinquecirrha*, *C*. *fuscescens*, *C*. *achlyos*, *Phyllorhiza* sp., and *Cephea cephea*. Dr. Garhard Jarms, previously at University of Hamburg, provided *Mastigias papua*, *Carybdea* sp. and the southern Japan strain of *A*. *aurita*. Wyatt Patry of the Monterey Bay Aquarium provided a culture of the coronate *Linuche* sp. The company Blue Corner, with support from the New England Aquarium, provided *Aurelia* sp. from northern Japan. Dr. Paulyn Cartwright of the University of Kansas and Dr. Matthew Nicotra of University of Pittsburgh provided *Podocoryna carnea*. Dr. Paulyn Cartwright also provided *Craspedacusta sowerbii*.

We kept polyps at water temperatures reported in Tables 1, S8 and S9. We regularly fed all animals newly hatched *Artemia* sp. (brineshrimpdirect.com) and changed aquaria water as needed.

**Table 1:** Strobilation response for discomedusan and cubozoan polyps in the presence of either indomethacin or 5-methoxy-2-methylindole.

Compound	Experiment number	Polyps tested individually (i) or collectively (c)	Species (drug concentration)	Temperature (C)	Number of animals, experiment (control)	Mean days to first sign of medusa formation	Standard deviation days to first sign of medusa formation	Percent experimental animals that produced medusae	Percent control animals that produced medusae	P-value (Fisher's exact test)
Indomethacin	1	c	*Aurelia* sp. N. Japan line (50 μM)	22	4 (4)	9.00	NA	100%	0%	0.029
Indomethacin	5	c	*Aurelia* sp. S. Japan line (50 μM)	22	4 (4)	11.00	NA	100%	0%	0.029
Indomethacin	8	i	*Cassiopea* sp. (50 μM)	22	6 (7)	45.00	2.83	83%	0%	0.005
Indomethacin	10	i	*Cephea cephea* (50 μM)	26.7	10 (14)	20.44	6.25	100%	14%	<0.001
Indomethacin	12	i	*Chrysaora achlyos* (50 μM)	22	6 (6)	5.50	0.84	100%	0%	0.002
Indomethacin	6	c	*Chrysaora fuscescens* (50 μM)	18	4 (4)	7.00	NA	100%	0%	0.029
Indomethacin	2	c	*Chrysaora pacifica* (50 μM)	18	3 (3)	6.00	NA	100%	0%	0.100
Indomethacin	4	c	*Chrysaora quinquecirrha* (50 μM)	22	3 (3)	7.00	NA	100%	0%	0.100
Indomethacin	7	i	*Cotylorhiza tuberculata* (50 μM)	22	6 (6)	30.33	1.63	100%	0%	0.002
Indomethacin	24	i	*Cyanea* sp. Woods Hole (50 μM)	18	20 (20)	11.46	1.68	100%	50%	<0.001
Indomethacin	11	c	*Mastigias papua* (50 μM)	26.7	16 (16)	14.00	NA	100%	0%	<0.001
Indomethacin	9	i	*Phyllorhiza punctata* (50 μM)	22	6 (6)	26.67	5.47	100%	0%	0.002
5-methoxy-2-methylindole	15	i	*Aurelia* sp. S. Japan line (50 μM)	22	7 (6)	2.00	0.00	100%	0%	<0.001
5-methoxy-2-methylindole	21	i	*Carybdea* sp. (5 μM)	26.6–29	18 (14)	13.00	0.00	100%	0%	<0.001
5-methoxy-2-methylindole	14	i	*Chrysaora quinquecirrha* (50 μM)	22	6 (6)	3.00	0.00	100%	0%	0.002
5-methoxy-2-methylindole	13	i	*Cotylorhiza tuberculata* (50 μM)	22	3 (3)	13.30	0.58	100%	0%	0.100
5-methoxy-2-methylindole	23	i	*Cyanea* sp. Woods Hole line (50 μM)	18	12 (12)	3.34	1.07	100%	8%	<0.001
5-methoxy-2-methylindole	16	i	*Mastigias papua* (50 μM)	26.7	6 (6)	7.50	0.55	100%	0%	0.002

### Chemical incubation trials

For all drug trials, polyps from each species were placed in pre-washed six, twelve or twenty-four well plates with 1–7 mL 0.2 μm filtered water. In some trials, multiple polyps were placed in single wells (experiments 1–6, 11, 17; S1 Table), while in other trials polyps were placed in individual wells (experiments 7–10, 12–16, 18–22; S1 Table). *Linuche* sp. form loose polyp colonies, and *P*. *carnea* is a colonial species, thus for both species each well contained multiple polyps. Plates were covered loosely in Parafilm to reduce evaporation. Polyps of most species were kept in 33 parts per thousand (ppt) artificial seawater (Instant Ocean). *Craspedacusta sowerbii* is a freshwater hydrozoan, and was kept in spring water (Poland Springs). *Linuche* sp. polyps were kept at 37 ppt salinity, according to culturing instructions from the Monterey Bay Aquarium. All polyps were starved for 5–7 days prior to the experiment start date to preserve water quality, and kept in ambient light conditions (unchanged from pre-experiment conditions). Controls for all trials consisted of an equal or nearly equal (as noted; Table 1) number of polyps exposed to the carrier solutions without the test compounds. All controls and experimental trials were otherwise treated in the exact same manner over the course of the trials. We recorded the time of compound addition for each sample and treatment, made visual observations every 24 hours, and recorded any changes. Observations were made daily (S2, S3, S4, S5, S6 and S7 Tables). We reduced the observation frequency to every other day for trials that ran more than several weeks without change.

#### Indomethacin trial

We prepared a 50 mM indomethacin stock solution by adding 18 mg of powdered indomethacin (Sigma-Aldrich, I7378-25G) to 1 mL DMSO. We added indomethacin stock solution to animal media at a ratio of 1 μl stock to 1 ml media for a final concentration of 50 μM. Indomethacin rapidly precipitates in water, so we stirred each treatment to facilitate rapid dissolving/resuspension of indomethacin.

#### 5-methoxy-2-methylindole trial

We prepared stock solutions of 5-methoxy-2-methylindole (Sigma-Aldrich, M15451-5G) by adding 16 mg 5-methoxy-2-methylindole to 2 mL 100% ethanol, for a final concentration of 50 mM. We prepared working dilutions by adding 1 μL for every 1 mL medium, for a final concentration of 50 μM. We conducted dilutions of this final 50 μM to obtain 20 μM and 5 μM solutions. In the case of *Linuche* sp. and *Podocoryna carnea* trials the water quality visibly decreased, so we exchanged the solution mid-trial.

#### Signs of medusa production

For scyphozoans, two methods for measuring medusa production were used. For polydisc strobilation, we used time from drug addition until first sign of strobilation. In these trials, we considered the first sign of strobilation to be the formation of the first strobilation furrow underneath the polyp tentacles. We followed all animals through complete strobilation to ensure successful liberation of medusae. No animal initiated strobilation that did not liberate medusae. We did not record the time of medusa liberation in these animals because large strobilae often fractured during movement of the dishes, rather than through ephyra pulsing. For species that only produce one ephyra (know as monodisc strobilators) and the cubozoan, the first signs of metamorphosis are not easy to distinguish, and so we used time from drug addition until we observed free-swimming medusae. These species produced vigorously swimming medusae, and are much less susceptible to mechanical liberation from movement of the dishes. For monodisc strobilators, liberation was much more temporally synchronized than polydisc strobilators. For hydrozoans, we used the interval from drug addition until presence of medusae as a measure of medusa production (however, no medusae were ever produced).

#### Statistical analysis and tree topology

To examine the distribution of indole-induced strobilation responses in a phylogenetic context, we mapped the presence or absence of a strobilation response onto a topology of Medusozoa congruent with multiple phylogenetic analyses of the group [22–25].

We used Fisher's exact test to determine the significance of a drug response relative to controls (Table 1).

## Results

**Fig 1:**
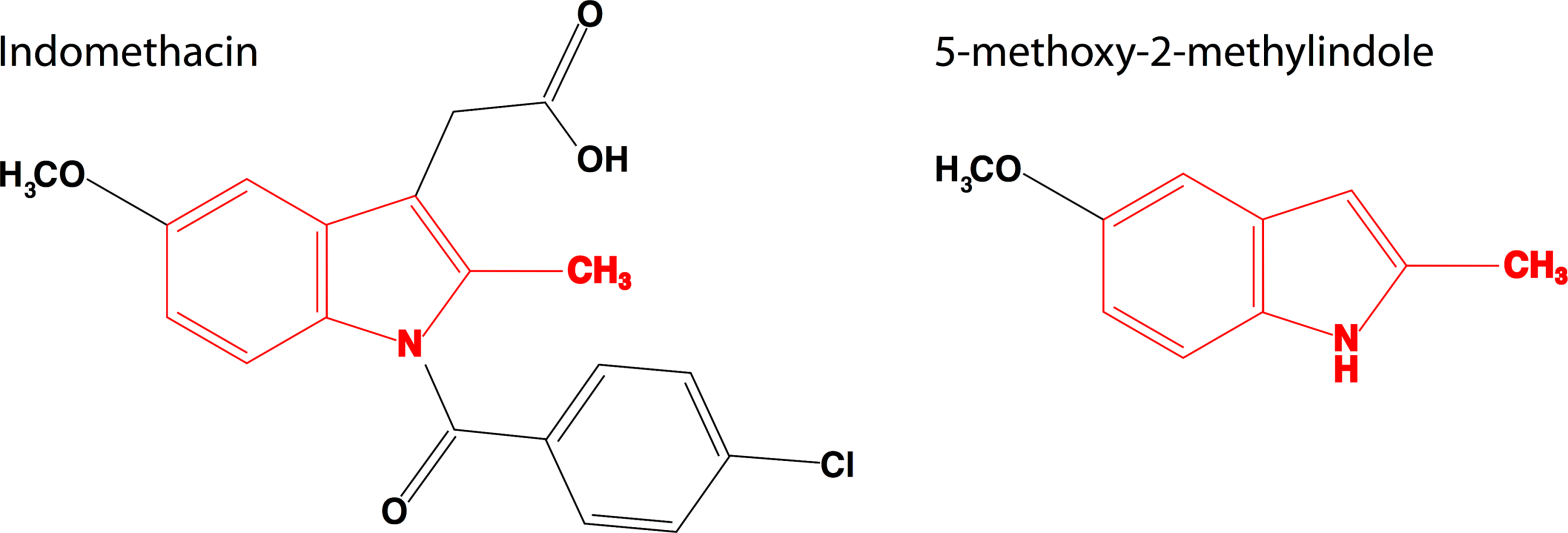
Chemical structures of indomethacin and 5-methoxy-2-methylindole, showing the conserved indole region in red.

**Fig 2:**
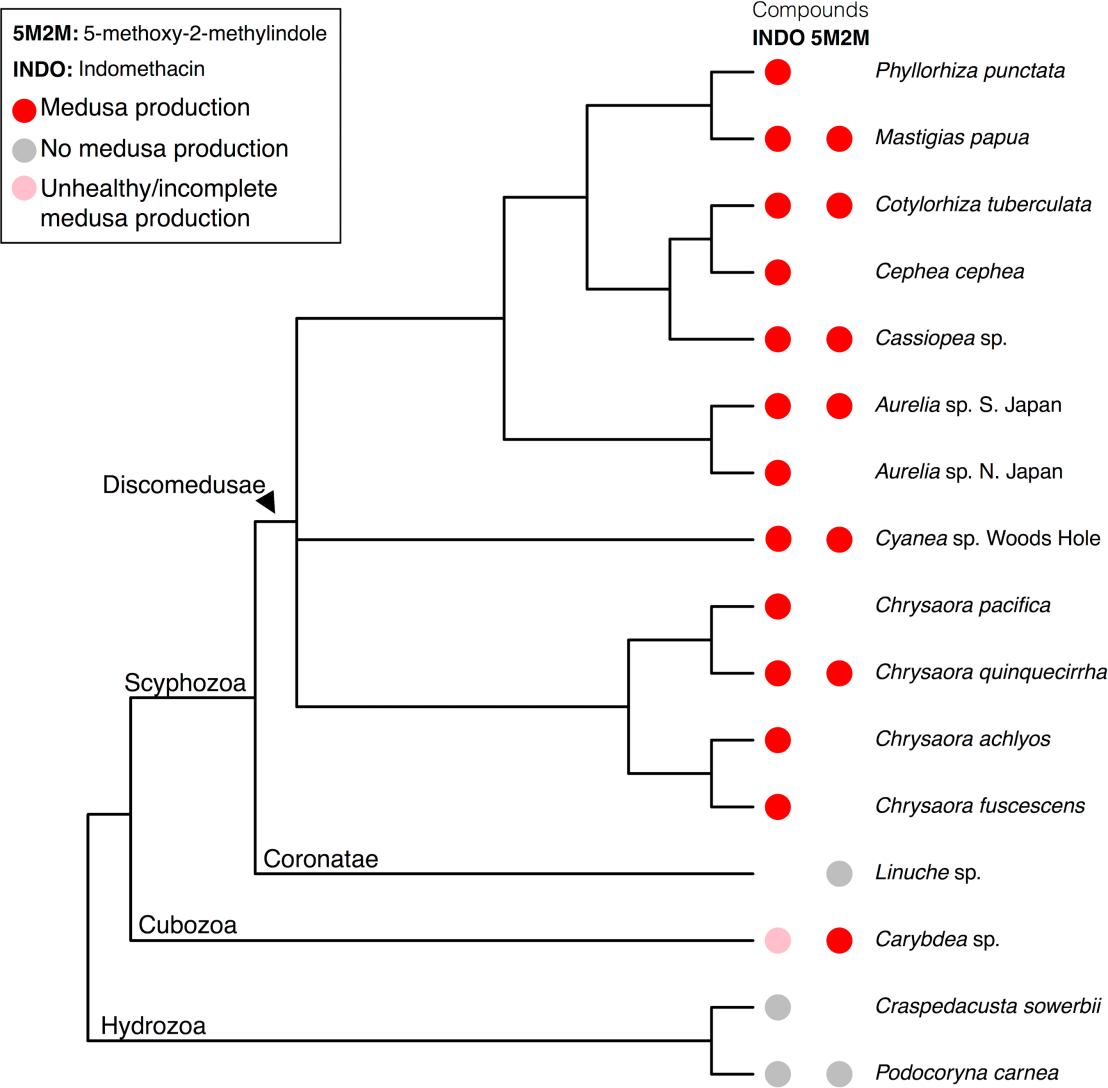
Polyps of Discomedusae and Cubozoa produce medusae (red circles) in the presence of some or all indoles, but polyps of the Coronatae or Hydrozoa do not (grey circles). Pink circles indicate a partial metamorphosis response. White space indicates that the experiment was not performed. The relationships between Hydrozoa, Cubozoa, and Scyphozoa is based on [23]. The topology is Scyphozoa is based on [24], except for the *Chrysaora* clade, which is based on [25].

**Fig 3:**
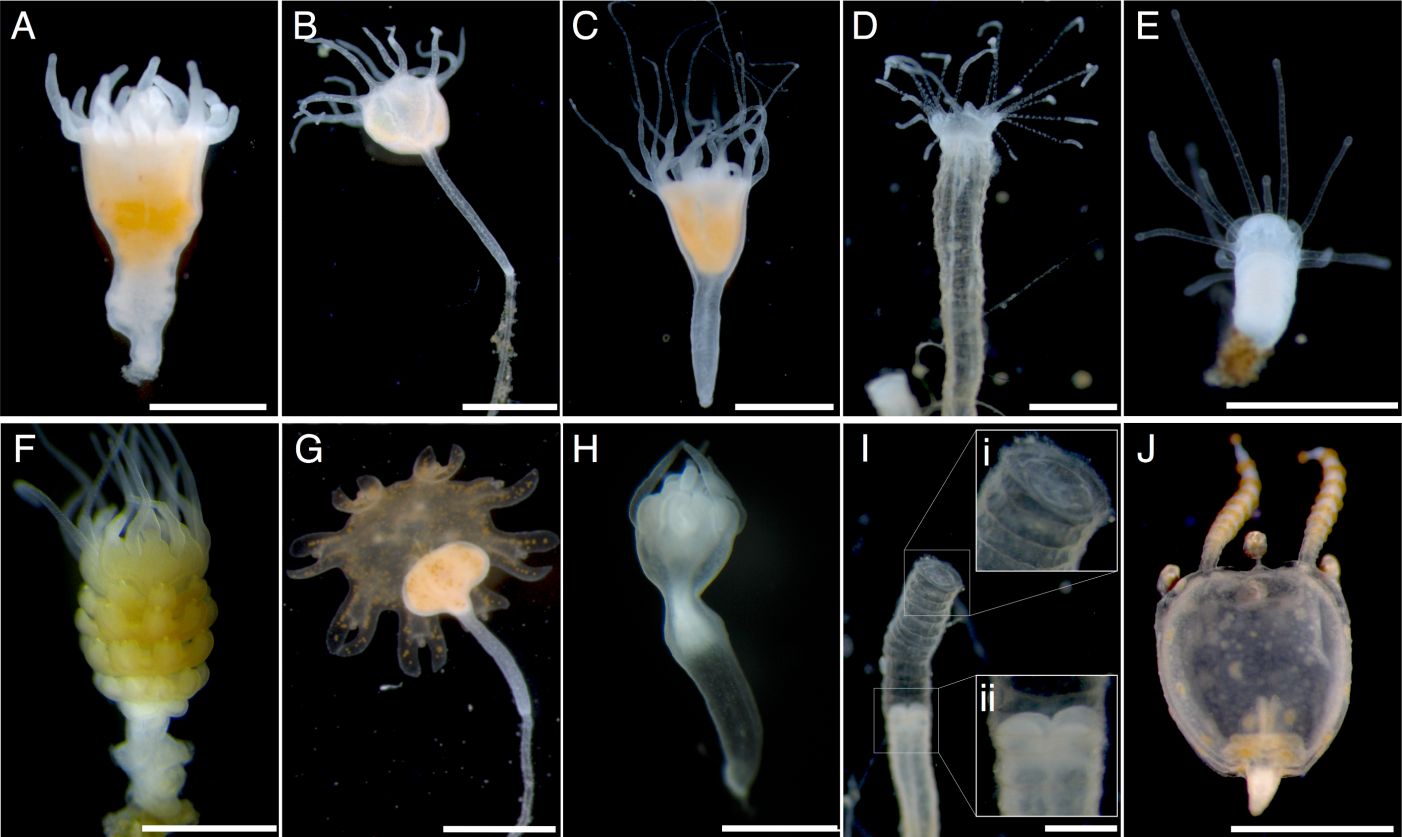
Indomethacin-induced strobilation in five species. All animals are oriented oral end facing up. Panels A-E are control polyps, panels F-J are animals exposed to indoles. A) *Aurelia aurita* polyp from southern Japan, and F) the same species showing classic polydisc strobilation, with each segment being a small immature ephyra. B) *Mastigias papua* polyp, and G) with classic monodisc strobilation, where the calyx slowly transforming into an ephyra. Note that the tissue below the calyx is not metamorphosing back into a polyp, which is typical of continuous exposure to indoles. C) *Cotylorhiza tuberculata* polyp, H) with monodisc strobilation, showing a constriction at the base of the calyx. D) a polyp of *Linuche* sp., showing classic coronate morphology in a chitinous tube, and I) in the presence of an indole, with a i) sealed operculum, and ii) retracted polyp. E) a polyp of the box jelly *Carybdea* sp., and J) the same species after metamorphosis into a small medusa, complete with a fragment of polyp tissue (at the apex of the bell). Scale bars = 1 mm.

Combined, we tested two indole-containing compounds, indomethacin and 5-methoxy-2-methylindole (Fig 1), on 16 medusozoan species. We found that both indomethacin and 5-methoxy-2-methylindole induce total and healthy metamorphosis in 12 species, 11 of which are from the monophyletic scyphozoan group Discomedusae (Figs 2 and 3). At low concentrations 5-methoxy-2-methylindole also induced metamorphosis in the cubozoan species *Carybdea* sp. Surprisingly, medusae were not produced in response to either drug for the coronate *Linuche* sp. Below, we present the results for each major clade.

### Indoles induced strobilation in all tested discomedusan polyps, but not a representative of the Coronatae

All tested species of Discomedusae strobilated in the presence of indomethacin (Fig 2), with response times ranging from two days to over one month (Table 1). This includes species from many of the major Scyphozoa lineages (Pelagiidae, Ulmaridae, Cyaneidae, Cepheidae, Cassiopeidae, and Mastigiidae), and polyps with both polydisc and monodisc strobilation (Fig 3). Because data for monodisc strobilators were collected using ephyra liberation as a measure of compound efficacy, the rate of strobilation for monodisc and polydisc species cannot be directly compared. However, within monodisc strobilators, the response of *Mastigias papua* to indomethacin was the most rapid, with ephyrae being liberated in just two weeks. In comparison, the monodisc strobilator *Cassiopea* sp. took 45 days.

We also tested 5-methoxy-2-methylindole on a subset of species, and observed a similar metamorphosis response to that observed with indomethacin, though with much more rapid response times (Table 1). For example, polyps of *C*. *quinquecirrha* strobilated three times faster, and *M*. *papua* ephyrae were liberated in less than half the time (Table 1).

Unlike discomedusan polyps, coronate polyps are entirely covered by a chitinous tube (Fig 3). We obtained a small number of polyps and, based on our previous results with discomedusan polyps, tested the effect of only 5-methoxy-2-methylindole in two concentrations: 50μM and 5 μM (S6 and S7 Tables). At the onset of naturally-induced strobilation, the polyp of *Linuche* sp. retracts and resorbs the oral disk [26]. In our exposure trials, polyps retracted the oral disk, and many polyps also formed a chitinous opercula in response to indole exposure (Fig 3). Unlike all tested discomedusan polyps, the coronate *Linuche* sp. did not strobilate in the presence of 5-methoxy-2-methylindole at either concentration. Instead, most *Linuche* sp. exhibited distinct morphological changes that did not clearly correspond to metamorphosis. By the end of both trials, significantly more experimental animals were retracted into their protective tubes, compared to controls (Fisher’s exact test, p < 0.01 for 50 μM concentrations, and p = 0.01 for 5 μM concentrations), this suggests a stress response, which may or may not have involved the initiation and cessation of strobilation.

### Drug response of the cubozoan *Carybdea* sp.

We tested polyps of *Carybdea* sp. under several different combinations of environmental and chemical concentration (Table 1). Lower concentrations of 5-methoxy-2-methylindole effectively induced healthy metamorphosis at 26.6°C-29°C. The best result was for 5 μM: 17 of 18 polyps produced healthy medusae in two weeks (with one polyp dying before the end of the trial). These medusae appeared morphologically normal, and exhibited normal swimming behavior (Fig 3).

However, polyps treated with indomethacin, or with higher doses of 5-methoxy-2-methylindole, exhibited only partial metamorphosis or other phenotypic changes (such as contracting into a ball, or tissue sloughing) that may be associated with stress or toxicity (S5, S9). Under these sub-optimal conditions, the hypostomes of experimental polyps developed into swollen rounded balls. In some cases the development of this swollen hypostome was accompanied by tentacle resorption and the formation of putative medusa rhopalial rudiments. However, most polyps did not complete metamorphosis, and eventually resorbed their rhopalial rudiments. In rare cases when medusae did develop, they were abnormal with wrinkled and diminished bells. From these results, we conclude that 50μM concentrations of either compound induces metamorphosis within *Carybdea* sp., but inconsistently and with severe adverse effects that may be related to toxicity.

Regardless of the outcome of metamorphosis, *Carybdea* sp. polyps incubated in 5-methoxy-2-methylindole responded faster to drug exposure than polyps incubated in indomethacin (Table 1). For example, polyps incubated in 20μM 5-methoxy-2-methylindole formed rhopalial rudiments and eye spots within six days. Polyps incubated in 20μM indomethacin formed eye spots only after 20 days (S9 table).

### Two hydrozoan species did not produce medusae in the presence of indoles

The hydrozoans *Podocoryna cernea* and *Craspedacusta sowerbii* did not produce medusae in the presence of 50μM indomethacin (Fig 2; S8 Table). In addition, *P*. *cernea* did not produce medusae in the presence of 50μM 5-methoxy-2-methylindole (*Craspedacusta sowerbii* was not tested; S8 Table). *Podocoryna cernea* and *C*. *sowerbii* treated with indomethacin showed no evidence of medusa production over the course of the experiments, though both control and experimental animals showed signs of stress after an extended period, possibly due to lack of food (S8 Table). *Podocoryna cernea* are colonial and form mats of polyps, however, polyps treated with 5-methoxy-2-methylindole failed to grow new mats or stolons, unlike control colonies (S8 Table).

### Constant exposure to indoles inhibits polyp reformation in Discomedusae

In naturally-induced medusa development in Discomedusae, a polyp reforms at the end of metamorphosis. Continuous indole exposure inhibits this response. When discomedusan polyps were continuously exposed to indomethacin or 5-methoxy-2-methylindole, strobilation proceeded until no tissue remained. After suitable amounts of tissue were no longer available, the ephyrae that formed were nothing more than small spheres with 1–3 rhopalia. In both *C*. *quinquecirrha* and *Aurelia* lines exposed to indomethacin, transient ectopic polyp tentacles occasionally formed below the last disk, and then quickly resorbed as a new ephyra disk formed. These observations suggest that polyp re-formation is initiated, but then quickly overridden, by the presence of exogenous indoles.

## Discussion

Our results support the hypothesis that there is a conserved molecular pathway involved in metamorphosis induction within Discomedusae [20], first observed in *Aurelia aurita* in 2012 [16]. Despite a long list of chemical and environmental cues known to induce metamorphosis in a species-specific manner within this clade [17,19,27–29], indomethacin and 5-methoxy-2-methylindole are the first compounds to induce metamorphosis across a broad diversity of species. The species we tested originate from a wide variety of habitats, ranging from tropical marine lakes to temperate northern coastal zones. In nature, each species likely develops medusae in response to radically different environmental triggers, yet all exhibit a conserved response to indole-containing compounds. This suggests that indoles may be targeting conserved core components of the cellular pathway involved in metamorphosis induction.

Our data also support previous findings that indoles induce metamorphosis in Cubozoa; A previous study also suggests this response is homologous with Scyphozoa [20]. This view is in contrast to the current textbook understanding of Cubozoa and Scyphozoa, which holds that each clade has a distinct mechanism of metamorphosis [8,9,30]. However, several recent studies suggest cubozoan metamorphosis may indeed possess highly modified strobilation [10–12].

However, our study is the first to include a coronate species. Coronatae occupies an important phylogenetic position as the sister group to Discomedusae [23]. Thus, in order to draw evolutionary comparisons between Scyphozoa and other lineages, it is important to sample from both Discomedusae and Coronatae. We discovered that 5-methoxy-2-methylindole does not induce metamorphosis in the coronate *Linuche* sp. This suggests several interesting alternative evolutionary scenarios for the origin of metamorphosis induction via indoles.

First, and simplest, differences in polyp morphology may have inhibited indole exposure or a complete drug response in *Linuche* sp. The unique morphology of coronate polyps stands in stark contrast to polyps of Discomedusae or Cubozoa. Coronate polyps are covered in a large chitinous tube that they retract into when startled. Most of the Coronate polyp body is covered by this tube, which may interfere with drug exposure. In support of this hypothesis, we observed significantly more contracted *Linuche* sp. polyps in experimental trials, compared to controls. We also observed chitinous opercula on experimental polyps but not on control polyps (Fig 3). In *Linuche* sp., the first stage of strobilation is the retraction of the polyp calyx [26]. The retraction we observed may in fact represent the first stages of strobilation, which then ceased due to inadequate drug exposure once the polyps formed opercula. However, some small polyps did not retract, and remained extended throughout the duration of trials, complicating this scenario.

Second, it is possible that *Linuche* sp. has lost this indole response, which may be related to its unique forms of strobilation. *Linuche* sp. can undergo a type of strobilation not observed in Discomedusae. In *Linuche unguiculata*, the top disc forms an ephyra, while lower discs form planuloids—round planula-like motile balls that swim out of the tube, settle, and form new polyps [26]. One *Linuche* sp. in our care strobilated naturally, and its strobilation was identical to that reported in *Linuche unguiculata*: one ephyra formed, and the remaining discs became planuloids. This unique form of strobilation—which forms multiple different types of bodies—may be accompanied by a unique form of strobilation induction, not present in Discomedusae, where only one type of body (the medusa) is formed. Combined, differences in polyp morphology and strobilation outcome may explain why indoles do not induce strobilation in a coronate species.

Third, it is possible that Cubozoa and Discomedusae independently evolved similar cellular mechanisms for inducing metamorphosis. Perhaps a similar evolutionary history associated with environmental cues may have resulted in the evolution of a convergent indole response between Discomedusae and Cubozoa. Testing a broader diversity of both Coronates and Cubozoans will help distinguish between these different hypotheses. Additionally, data from Staurozoa will provide important taxonomic insights into the efficacy of indoles in inducing metamorphosis in this clade, and the origin of indole-induced metamorphosis.

No tested hydrozoans produced medusae in the presence of indoles, which is consistent with previous findings [20]. In contrast to either cubozoan or scyphozoan polyps, hydrozoan polyps produce medusae through budding [4], which may occur continuously under conducive environmental conditions. Hydrozoans’ developmentally distinct process of medusae formation may be triggered by fundamentally different molecular pathways than those that trigger strobilation in cubozoans and scyphozoans.

The evolutionarily conserved response of polyps of Discomedusae to indoles hints at a conserved molecular pathway for triggering strobilation across diverse species. Several candidate pathways are ideal targets to examine first. In vertebrates, indomethacin is an agonist of the nuclear hormone receptor Peroxisome Proliferator-Activated Receptor (PPAR) [31], which forms heterodimers with another nuclear receptor, retinoid X receptor (RxR). While no clear homologues of PPAR have been found in the sea anemone *Nematostella vectensis* [32], the presence or absence of PPAR in scyphozoans and cubozoans is unknown. In contrast, bona fide homologues of RxR are present in Discomedusae, Cubozoa, and Hydrozoa [33]. RxR transcripts are upregulated during strobilation in the Discomedusae *Aurelia* sp., and the receptor has been implicated in the onset of metamorphosis induction [17]. Nuclear hormone receptors may be involved in the induction of metamorphosis in Cubozoa and Scyphozoa, and their role in all medusozoans (including hydrozoans) deserves further investigation.

It is unknown if there are endogenous indole compounds produced by polyps that trigger metamorphosis. Fuchs [17] identified a small peptide sequence (WSRRRWL) from *Aurelia* sp. 1, with two indole-containing tryptophans, that induces strobilation (L-tryptophan alone does not induce strobilation in this line [17]). This peptide is derived from a gene (CL390) whose transcript is upregulated during naturally-induced strobilation, and Fuchs *et al*. hypothesize that it may be the source of indoles. However, they also note that this peptide does not induce metamorphosis in other *Aurelia* lines. A later transcriptomics study by Brekhman *et al*. [34] through the full life cycle of an *Aurelia aurita* from the Red Sea recovered a potential CL390 homologue, but it is missing the WSRRRWL peptide sequence. Indoles may be derived from another biological pathway, sensed from external sources, or synthetic indoles may be acting as agonists/antagonists for receptors that bind substrates other than indoles.

## Conclusions

We demonstrate that indole compounds induce metamorphosis in an evolutionarily conserved manner, but raise questions about how well this is conserved beyond a subclade of Scyphozoa. Indoles induced metamorphosis in every tested discomedusan polyp, and a cubozoan species, yet did not induce metamorphosis in the coronate *Linuche* sp. If indole induction is homologous between Discomedusae and Cubozoa, it has been lost in *Linuche* sp. Otherwise, induction is a convergent features of Discomedusae and Cubozoa. Discovering the molecular target(s) of indoles will help distinguish between these two hypotheses. Comparative transcriptomic studies in diverse species after indole exposure will also help determine if patters of gene expression are consistent with evolutionarily conserved responses [35].

## Supporting information

S1 Table Experimental key. This file contains a key to all experiments performed and location of the raw data within the supplementary files. This is a PDF file.
(PDF)

S2 Table Raw data for scyphozoans exposed to 5-methoxy-2-methylindole. This file is presented in tidy data format, and contains strobilation data for the following species: *Cotylorhiza tuberculate*, the *Aurelia* sp. S. Japan line, *Mastigias papua*, and *Cyanea* sp. Woods Hole line. This is a tab-separated text file.
(TSV)

S3 Table Raw data for species exposed to indomethacin in trials where multiple animals were pooled per treatment. This file is presented in tidy data format, and contains strobilation data for the following species: *Aurelia* N. Japan line, *Chrysaora pacifica*, *Chrysaora quinquecirrha*, *Aurelia* sp. S. Japan line, *Chrysaora fuscescens*, and *Mastigias papua*. This is a comma-separated text file.
(CSV)

S4 Table Raw data for species exposed to indomethacin in trials where treatments consisted of individual animals. This file is presented in tidy data format, and contains strobilation data for the following species: *Cotylorhiza tuberculate*, *Cassiopea* sp., *Phyllorhiza punctate*, *Cephea cephea*, *Chrysaora achlyos*, and *Cyanea* sp. Woods Hole. This is a tab-separated text file.
(TXT)

S5 Table Raw data for *Carybdea* sp. exposed to various concentrations of 5-methoxy-2-methylindole. This is a tab-separated text file in tidy data format.
(TSV)

S6 Table Raw data for *Linuche* sp. exposed to 5μM 5-methoxy-2-methylindole in trials where multiple replicates of multiple pooled animals were exposed to the drug or control. This is a tab-separated text file in tidy data format.
(TSV)

S7 Table Raw data for *Linuche* sp. exposed to 50μM 5-methoxy-2-methylindole in trials where single replicates of multiple pooled animals were pooled per treatment. This is a tab-separated text file in tidy data format.
(TSV)

S8 Table Summary table for trials where there was no metamorphosis response. This is a TSV file.
(TSV)

S9 Table Summary table for trials for *Carybdea* sp. where there was a partial metamorphosis response. This is a TSV file.
(TSV)
