# Supplementary material for: Indoles induce metamorphosis in a broad diversity of jellyfish, but not in a crown jelly (Coronatae)
Source: PLoS One. 2017 Dec 27;12(12):e0188601. doi: 10.1371/journal.pone.0188601 (PMC5744923; doi:10.1371/journal.pone.0188601)
Supplement: S1 Table — This file contains a key to all experiments performed and location of the raw data within the supplementary files. This is a PDF file. (PDF) [file pone.0188601.s001.pdf]

Experiment	Species	compound	concentration (μM)	year	spreadsheet
1	*Aurelia* sp. N. Japan	indomethacin	50	2013	S3 Table
2	*Chrysaora pacifica*	indomethacin	50	2013	S3 Table
3	*Craspedacusta sowerbii*	indomethacin	50	2013	S8 Table
4	*Chrysaora quinquecirrha*	indomethacin	50	2013	S3 Table
5	*Aurelia* sp. S. Japan	indomethacin	50	2013	S3 Table
6	*Chrysaora fuscescens*	indomethacin	50	2013	S3 Table
7	*Cotylorhiza tuberculata*	indomethacin	50	2015	S4 Table
8	*Cassiopea* sp.	indomethacin	50	2015	S4 Table
9	*Phyllorhiza punctata*	indomethacin	50	2015	S4 Table
10	*Cephea cephea*	indomethacin	50	2015	S4 Table
11	*Mastigias papua*	indomethacin	50	2015	S3 Table
12	*Chrysaora achlyos*	indomethacin	50	2015	S4 Table
13	*Cotylorhiza tuberculata*	5-methoxy-2-methylindole	50	2015	S2 Table
14	*Chrysaora quinquecirrha*	5-methoxy-2-methylindole	50	2015	S2 Table
15	*Aurelia* sp. S. Japan	5-methoxy-2-methylindole	50	2015	S2 Table
16	*Mastigias papua*	5-methoxy-2-methylindole	50	2015	S2 Table
17	*Linuche* sp.	5-methoxy-2-methylindole	5	2016	S6 Table
18	*Linuche* sp.	5-methoxy-2-methylindole	50	2016	S7 Table
19	*Carybdea* sp.	5-methoxy-2-methylindole	50	2016	S5 Table
20	*Carybdea* sp.	5-methoxy-2-methylindole	20	2016	S5 Table
21	*Carybdea* sp.	5-methoxy-2-methylindole	5	2016	S5 Table
22	*Carybdea* sp.	Indomethacin	50, 20, 5, 0	2016	S9 Table
23	*Cyanea* sp. Woods Hole	5-methoxy-2-methylindole	50	2016	S2 Table
24	*Cyanea* sp. Woods Hole	Indomethacin	50	2017	S3 Table
